# Cost-of-illness trajectories among people with multiple sclerosis by comorbidity: A register-based prospective study in Sweden

**DOI:** 10.1177/2055217320968597

**Published:** 2020-10-23

**Authors:** Greta Bütepage, Ahmed Esawi, Kristina Alexanderson, Emilie Friberg, Chantelle Murley, Jan Hillert, Korinna Karampampa

**Affiliations:** Division of Insurance Medicine, Department of Clinical Neuroscience, Karolinska Institutet, Stockholm, Sweden; Division of Neurology, Department of Clinical Neuroscience, Karolinska Institutet, Stockholm, Sweden; Division of Insurance Medicine, Department of Clinical Neuroscience, Karolinska Institutet, Stockholm, Sweden

**Keywords:** Multiple sclerosis, cost-of-illness, comorbidity, sick leave, healthcare costs, productivity losses, trajectories

## Abstract

**Background:**

Comorbidities are common among people with multiple sclerosis (PwMS); yet, their impact on the cost-of-illness (COI) in MS is unknown.

**Objective:**

Explore the heterogeneity in COI trajectories among newly diagnosed PwMS in relation to type of comorbidity.

**Methods:**

A nationwide longitudinal cohort study, using prospectively collected Swedish register data for seven years. The COI/year of 639 PwMS diagnosed in 2006, when aged 25–60, was estimated until 2013. Using healthcare data, PwMS were categorised into six comorbidity groups: ocular; cardiovascular, genitourinary or cancer disease; musculoskeletal; mental; neurological other than MS; and injuries. One group of PwMS without comorbidity was also created. Group-based trajectory modelling was applied, examining different COI trajectories within each comorbidity group.

**Results:**

Across the seven follow-up years, PwMS with mental comorbidities had the highest COI overall (€36,482). Four COI trajectories were identified within each comorbidity group; the largest trajectory had high healthcare costs and productivity losses (36.3%–59.6% of PwMS, across all comorbidity groups). 59.6% of PwMS with mental comorbidity had high healthcare costs and productivity losses.

**Conclusion:**

High COI and heterogeneity in COI trajectories could be partly explained by the presence of chronic comorbidities in the year around MS diagnosis, including the presence of mental comorbidity.

## Introduction

Multiple sclerosis (MS) is a progressive neurological disease and the most common cause of neurological disability in young adults.^1,2^ Typically diagnosed when aged 20-40,^1^ MS leads to variable degrees of physical and cognitive impairment, and can result in significant work incapacity and healthcare resource use.^1^ In 2010, the estimated cost of illness (COI) of MS in Sweden was €414 million.^3^

People with MS (PwMS) have a high rate of comorbidity, including both mental (e.g. depression and anxiety) and somatic (e.g. hypertension, diabetes) conditions.^4^

Diagnostic delay, high level of disability at diagnosis, and worse clinical outcomes are some of the clinical implications of comorbidity among PwMS.^5,6^ Compared with matched reference groups from the general population, PwMS with comorbidity have a higher risk of hospitalisation and physician visits in primary care.^7,8^ In addition, comorbidity is linked with higher sickness absence (SA) and disability pension (DP) in PwMS.^3,4^

A recent Swedish study showed that PwMS follow different COI trajectories.^9^ Utilising information about prescribed drugs to capture the presence of comorbidity in PwMS at the time of diagnosis, comorbidity was found to be associated with high levels of healthcare costs and productivity losses over time.^9^ Yet, ’alternative methods for identifying comorbidities at the time of diagnosis are needed to further deepen the knowledge of associations with COI.

Understanding the link between comorbidity and the heterogeneity in the development of COI of MS is paramount to optimise patient care and healthcare services for PwMS. Therefore, this study aimed to explore the heterogeneity in COI trajectories among newly diagnosed PwMS in relation to the type of comorbidity at the time around diagnosis, and to analyse the socio-demographic composition of each identified trajectory group.

## Methods

### Study design and data collection

A register-based cohort study was conducted, with data collected prospectively from five nationwide registers, linked using the unique personal identity numbers issued to all residents in Sweden.^10,11^ Data are kept by the following authorities:

• National Board of Health and Welfare:
1. The Swedish National Patient Register (NPR): inpatient and specialised outpatient healthcare data, including dates and diagnoses
2. The Swedish Prescribed Drug Register (SPDR): dates, names, and costs of dispensed prescription medications
3. The Cause of Death Register: year of death
• Statistics Sweden:
1. The Longitudinal Integration Database for Health Insurance and Labour Market Studies (LISA): socio-demographic variables in 2006 (sex, age, marital status, educational level, type of living area, birth country)
• Swedish Social Insurance Agency:
2. Micro Data for Analysis of Social Insurance (MiDAS): start and end dates, and grades (full- or part-time) of SA and DP

The study population (n = 793) was identified from the NPR and included all PwMS who had their first MS diagnosis (main or a secondary diagnosis) in 2006 when aged 25-60 years and who were alive and in Sweden in 2006, according to LISA. They were followed prospectively for seven years in the registers, from date of first MS diagnosis in 2006; censoring occurred at the date of death or the year of migration from Sweden.

Information about diagnoses for inpatient and outpatient healthcare (both main and secondary diagnoses) was used to identify comorbid conditions within 6 months before and after the MS diagnosis date, i.e., during a 12-month period. Six comorbidity groups were compiled from the cohort according to the chapters of the International Classification of Disease, Tenth Revision (ICD-10) codes:^12^ ocular (H00-H59); cardiovascular, genitourinary, or cancer disease (I00-I99, N00-N99, C00-D49), musculoskeletal (M00-M99); mental (F30-48, F50-59); neurological other than MS (G00-G99, excluding G35 for MS); and injuries (S00-T88). The grouping was guided by the frequency of comorbid diagnoses in the study population, known association with MS, and previous investigations of the prevalence of comorbidities among PwMS.^3,4,13^ An individual could be included in more than one of these six groups, if having different types of comorbidity. A seventh group included PwMS without other diagnoses, i.e., PwMS without any comorbidity either in the six other comorbidity groups or any other diagnosis. Due to small numbers of people in the cohort with other specific diagnoses (<10 people in most cases) which were mainly not associated with MS, results for PwMS with those other diagnoses were excluded and not reported in this study (n = 154). Therefore, results for n = 639 PwMS in total (analysis population/study cohort) are reported in this study, which comprises of PwMS belonging to any of the six comorbidity groups (n = 499) and PwMS without any comorbidity (n = 140).

### Study outcomes

Adopting a societal perspective, the estimated COI included both healthcare costs and productivity losses.^14^ All unit costs (Table 1) were inflated to 2018 prices and converted to Euros.^15,16^

**Table 1.** Unit costs used in the calculation of healthcare costs and productivity losses.

		Year	Value in SEK (2018)	Value in Euro (2018)	Source
Average inpatient and specialised outpatient healthcare cost per DRG		2006	44,355	5,014	Swedish Association for Local Authorities and Regions-Cost per patient for somatic morbidity^17^
		2007	44,838	5,007	
		2008	45,771	4,984	
		2009	46,329	4,967	
		2010	46,521	4,910	
		2011	46,797	4,882	
		2012	47,663	4,932	
		2013	50,260	5,180	
Co-payment for hospital stay (cost per day of stay)		2018	100	10	This was calculated at 100 SEK per day as this is the case for the majority of regions in Sweden. The max co-payment amount for inpatient healthcare was set to 1,500 SEK. Assumed for all Sweden based on information from the region Västra Götalandsregionen.
Co-payment for specialised outpatient healthcare (cost per visit)		2018	273	27	The max co-payment amount for specialised outpatient care was set to 1,100 SEK as only one region in Sweden had a ceiling value below 1,100 SEK.^33^
Monthly salary including employer contribution	Men	2006	41,817	4,076	The average monthly salary for men and women available from Statistics Sweden^35^ and multiplied by employer contribution rates obtained from the Swedish Tax Authority Sweden.^20^
		2007	42,421	4,135	
		2008	42,981	4,190	
		2009	43,907	4,280	
		2010	43,907	4,280	
		2011	43,768	4,267	
		2012	44,602	4,348	
		2013	45,713	4,456	
	Women	2006	35,244	3,436	
		2007	35,561	3,467	
		2008	36,121	3,521	
		2009	37,377	3,644	
		2010	37,654	3,671	
		2011	37,515	3,657	
		2012	38,349	3,738	
		2013	39,461	3,847	

Annual healthcare costs included inpatient and outpatient healthcare and dispensed prescribed drugs. Annual costs for such drugs were obtained from the SPDR. Inpatient costs were obtained by multiplying nationwide weights of diagnostic-related groups (DRG) by the average cost per 1.0 DRG and the number of hospitalisation days per patient.^3^ Outpatient costs were similarly calculated using specific DRG costs and multiplying by the number of specialised outpatient visits. Co-payments were calculated as the sum of patient fees for inpatient admissions and outpatient visits every year. Threshold values were applied if co-payments exceeded the current annual high-cost ceilings for out-of-pocket spending in inpatient and outpatient care (1500 SEK and 1100 SEK, respectively).

Annual productivity losses were estimated from the net days of SA and DP. Residents in Sweden with income from work or unemployment benefits are eligible for SA in the case of work incapacity due to disease or injury from the age of 16.^17^ The first day of a SA spell is a qualifying day with 100% loss of income. Employers reimburse lost income from day 2 to 14. From day 15 onwards, SA benefits are paid by the Swedish Social Insurance Agency, or from day 2 for those on unemployment benefits.^17^ To avoid bias due to employment status, only SA spells >14 days were included in the analysis. All people aged 19-64 can be granted DP, in the case of long-term or permanent work incapacity due to disease or injury.^17^ Both SA and DP can be granted for full-time (100%) or part-time (25%, 50%, or 75%) of ordinary work hours.^17^ Thus, individuals can be on part-time SA and DP simultaneously. Therefore, we calculated net absence days, e.g., two absence days on 50% was counted as one net day.

Pursuant to the human capital approach,^18^ productivity losses were computed by multiplying the net days on SA or DP with the average monthly salary per day, adding annual employer social insurance contributions (Table 1).^19^

### Analyses

Descriptive statistics for population characteristics and average annual COI per patient were calculated for each comorbidity group. Confidence intervals were generated by bootstrapping (1000 iterations).^20^

Healthcare costs and productivity losses were then ranked and categorised into quintiles, representing the relative levels of these costs, within each comorbidity group (the first quintile represented the lowest cost estimations; the fifth quintile represented the highest). Using these quintiles, group-based trajectory modelling (GBTM), using a quadratic, zero-inflated Poisson model^21^ was conducted in SAS® (Proc Traj)^22^ to identify clusters of individuals following similar trajectories of healthcare costs and productivity losses among PwMS with or without comorbidity and within the six individual comorbidity groups.^21^

Number of trajectories was decided based on: 1) knowledge of the observed COI trends using descriptive statistics, 2) size of trajectories (the size of each trajectory group) (>5%), 3) difference in the Bayesian information criterion (ΔBIC) – when testing models with an increasing number of trajectories, the model with the highest 2*ΔBIC was selected, 4) highest average posterior probabilities for belonging in the trajectory (>0.7), and 5) odds of correct classification (>5).

Due to the small sample size per comorbidity subgroup, no covariates (sociodemographic characteristics) were included into the GBTM. Instead, Pearson’s Chi-square and Fisher’s exact tests were used to analyse the socio-demographic differences across PwMS following different COI trajectories.^23^

The project was approved by the Regional Ethical Review Board of Stockholm.

## Results

The sociodemographic characteristics of the study cohort (n = 639) are presented in Table 2.

**Table 2.** Baseline socio-demographic characteristics by comorbidity group^a^ for people with MS, diagnosed in 2006 when aged 25–60 years.

	Any comorbidity	No comorbidity	Ocular comorbidities (H00-H59)	Cardiovascular, genitourinary or cancer disease (I00–I99, N00–N99, C00–D49)	Musculoskeletal comorbidities (M00–M99)	Mental comorbidities (F30–48, F50–59)	Neurological comorbidities (G00–G99)	Injuries (S00–T88)
	N = 499	N = 140	N = 169	N = 125	N = 74	N = 41	N = 238	N = 52
	n (%)	n (%)	n (%)	n (%)	n (%)	n (%)	n (%)	n (%)
**Sex**								
Men	89 (17.84)	57 (40.71)	50 (29.59)	31 (24.80)	16 (21.62)	19 (46.34)	81 (34.03)	11 (21.15)
Women	410 (82.16)	83 (59.29)	119 (70.41)	94 (75.20)	58 (78.38)	22 (53.66)	157 (65.97)	41 (78.85)
**Age at MS diagnosis**								
25–34	138 (27.66)	30 (21.43)	58 (34.32)	30 (24.00)	16 (21.62)	10 (24.39)	65 (27.31)	13 (25.00)
35–44	179 (35.87)	43 (30.71)	61 (36.09)	34 (27.20)	20 (27.03)	<10	69 (28.99)	14 (26.92)
45–54	120 (24.05)	37 (26.43)	33 (19.53)	40 (32.00)	16 (21.62)	14 (34.15)	73 (30.67)	17 (32.69)
55–60	62 (12.42)	30 (21.43)	17 (10.06)	21 (16.80)	22 (29.73)	<10	31 (13.03)	<10
**Educational level (years)**								
Elementary school (≤9)	57 (11.54)	17 (12.32)	18 (10.78)	16 (13.11)	10 (13.51)	<10	26 (11.06)	11 (21.15)
High school (10–12)	245 (49.60)	69 (50.00)	83 (49.7)	71 (58.20)	40 (54.05)	21 (53.85)	122 (51.91)	26 (50.00)
University/College (>12)	192 (38.87)	52 (37.68)	66 (39.52)	35 (28.69)	24 (32.43)	11 (28.21)	87 (37.02)	15 (28.85)
**Country of birth**								
Sweden	457 (92.51)	123 (89.13)	150 (89.82)	109 (89.34)	68 (91.89)	36 (92.31)	213 (90.64)	47 (90.38)
Nordic countries (except Sweden)	<10	()	<10	<10	<10	<10	<10	<10
EU27 (except Denmark, Finland, and Sweden)	10 (2.02)	<10	<10	<10	<10	()	<10	()
Rest of the world	22 (4.45)	11 (7.97)	11 (6.59)	<10	<10	<10	14 (5.96)	<10
**Type of living area**								
Big cities	189 (38.26)	54 (39.13)	70 (41.92)	51 (41.80)	30 (40.54)	13 (33.33)	105 (44.68)	20 (38.46)
Medium sized cities	169 (34.21)	49 (35.51)	55 (32.93)	38 (31.15)	26 (35.14)	12 (30.77)	66 (28.09)	15 (28.85)
Small towns/villages	136 (27.53)	35 (25.36)	42 (25.15)	33 (27.05)	18 (24.32)	14 (35.90)	64 (27.23)	17 (32.69)
**Family situation**								
Married or cohabitating without children at home	71 (14.37)	22 (15.83)	23 (13.77)	15 (12.30)	12 (16.22)	<10	42 (17.87)	<10
Married or cohabitating with children at home	203 (41.09)	61 (43.88)	71 (42.51)	43 (35.25)	27 (36.49)	10 (25.64)	88 (37.45)	16 (30.77)
Single without children at home	158 (31.98)	36 (25.9)	61 (36.53)	49 (40.16)	25 (33.78)	23 (58.97)	83 (35.32)	18 (34.62)
Single with children at home	62 (12.55)	20 (14.39)	12 (7.19)	15 (12.30)	10 (13.51)	<10	22 (9.36)	<10

^a^From people with MS that met the inclusion criteria for our study, only 499 of those had at least one relevant comorbidity at the time of MS diagnosis, i.e., they were belonging in at least one of the defined six comorbidity groups, or had no comorbidity (n = 140). Therefore, results in this table are presented for the analysis population of this study, i.e. for n = 639 people with MS. Since PwMS can present with multiple comorbidities, they can be included in more than one comorbidity group (out of these six comorbidity groups).

Pooled together, PwMS with any of the six comorbidities had an average annual COI of €25,930 per patient over the course of the study (see also the Online supplementary material, Tables 1 and 2,  Online supplementary material). PwMS with mental comorbidities had the highest average annual COI (€36,482). Conversely, PwMS without comorbidity in the year around the MS diagnosis had a lower average annual COI than any of the comorbidity groups (€22,465). The main cost driver was DP, irrespective of type of comorbidity.

**Figure 1.**
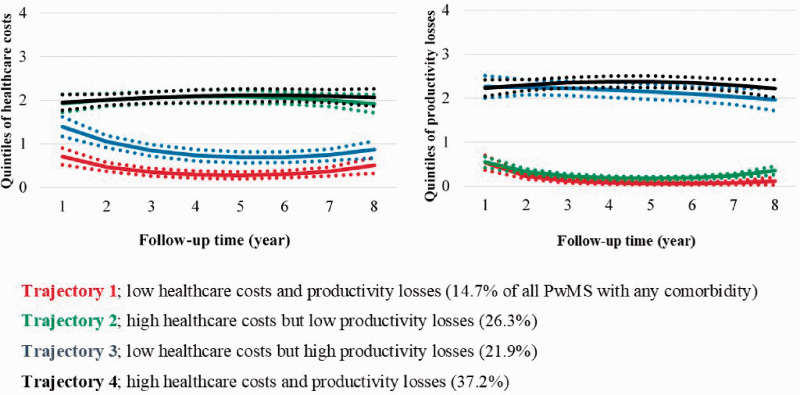
Trajectories of healthcare costs (HC) and productivity losses (PL), respectively, among the 499 people with MS who had any comorbidity (dotted lines represent 95% confident intervals), over the future seven years from date when diagnosed with MS in 2006.

Except for PwMS with injuries, the average healthcare costs and productivity losses increased in the beginning of follow-up and then decreased for most groups (Figure 1, Online supplementary material). The largest net decrease was observable in PwMS with musculoskeletal comorbidities (25.6%).

**Figure 2.**
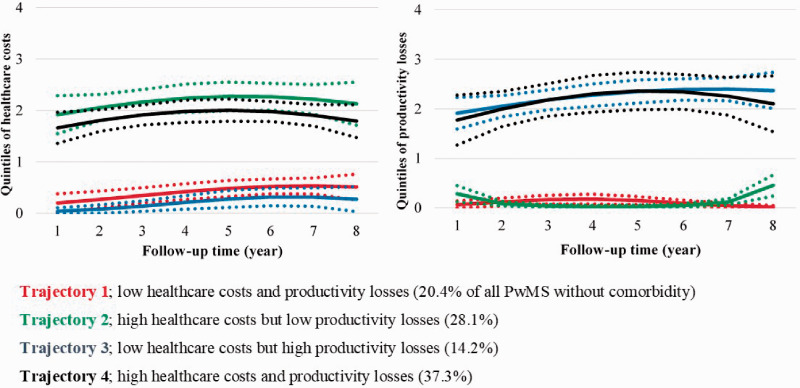
Trajectories of healthcare costs (HC) and productivity losses (PL), respectively, among the 140 people with MS but without comorbidities (dotted lines represent 95% confident intervals), over the future seven years from date when diagnosed with MS in 2006.

Four distinct COI trajectories were identified for healthcare costs and productivity losses (Figure 1) for the group of PwMS with any comorbidity (Figure 1), for those without any comorbidity (Figure 2), and for the six comorbidity groups (see Online supplementary material).

Irrespective of the presence of comorbidities, most PwMS belonged to the trajectory of high healthcare costs and productivity losses. The greatest proportions with high COI trajectories were observed among PwMS with mental comorbidities (59.6%), injuries (54.6%), and musculoskeletal comorbidities (50.2%); see also the Online supplementary material. Whereas only 37.3% of PwMS had high healthcare costs and productivity losses among those without comorbidity (Figure 2).

The sociodemographic composition of PwMS following the distinct trajectories differed across the comorbidity groups by age, educational level, and sex. PwMS with mental or neurological comorbidities who followed trajectories with high productivity losses were significantly older and had a lower educational level (at most high school education) (p < 0.05). The latter observation was also made for PwMS with cardiovascular, genitourinary, or cancer disease who sustained high productivity losses (p < 0.05). Sex was only significantly associated with the development of COI for PwMS with neurological comorbidities, where more women followed trajectories with high productivity losses (p < 0.05). Among PwMS without comorbidity, age and living area were significantly associated with the cost trajectories. Those with high productivity losses were significantly older and lived in small or medium sized cities (p < 0.05).

## Discussion

This register-based prospective cohort study was the first to explore the impact of different types of comorbidities present at MS diagnosis on their future COI trajectories among PwMS. The development of COI was heterogeneous, with the differences most apparent in the distribution of PwMS following specific COI trajectories across the comorbidity groups.

The proportion of PwMS found in the high healthcare costs and productivity losses trajectory ranged from 36.3 to 59.6%, with the highest proportions being associated with mental and musculoskeletal disorders, and with, to a lesser degree, injuries. This is in line with a previous study showing similar results.^3^ The large proportion of PwMS with injuries and high levels of healthcare costs and productivity losses could be interpreted as a consequence of MS and the disability can impose on individuals, increasing their risk of recurrent falling, which have previously been reported for PwMS who experienced injuries due to falls.^24^ In fact, high losses of productivity and increased resource use^3^ have previously been reported for PwMS with musculoskeletal comorbidities and injuries. Still, this result should be interpreted with caution. It is unknown whether injuries after diagnosed with MS may have a long-term impact on health of PwMS and hence, explain the high levels of healthcare costs and productivity losses observed.

Consistent also with previous results that mental disorders were among the diagnosis groups associated with the highest costs in PwMS in Sweden,^3^ the PwMS with mental comorbidities had the highest average annual COI per patient during our study. Still, only 5% of PwMS were found to have mental comorbidities - a percentage that can be considered low given that it is known that mental comorbidities are highly prevalent in the MS population,^25^ and also that the prevalence of major depression or generalized anxiety disorder in Sweden among adults in the general population is around 17%.^26^ This low proportion of mental comorbidity could be explained by the fact that we measured comorbidities at baseline (at diagnosis) and no follow-up of the presence of comorbidities over time was performed. Another reason for this is, of course, that we base the occurrence of comorbidity on data from inpatient or specialized outpatient healthcare, and not from primary healthcare. In Sweden, the first line treatment of mental disorders is handled by primary healthcare. Thus, we only captured the more severe mental disorders by this method, something that can be seen as both a strength and a limitation. Still, mental comorbidity at the time of MS diagnosis, is associated with high healthcare consumption, disability progression, and loss in productivity in PwMS,^3,6,7,27^ which is consistent with the large proportion of PwMS with mental comorbidities following the trajectory with high healthcare costs and productivity losses in our study.

By contrast, PwMS without comorbidity had the lowest average annual COI per patient overall. This difference could be related to the severity of the comorbidities, but to an extent, it is also driven by the misattribution of MS symptoms to other pre-existing conditions and non-observed comorbidities in this study. In fact, the high frequency of neurological comorbidities, and, to some degree, ocular and musculoskeletal comorbidities could be due to symptoms associated with MS prior to setting correct diagnosis. Another explanation of this difference is that the adverse health effects of certain comorbidities are assumed to contribute to a delayed MS diagnosis and a worse degree in disability at the time of MS diagnosis.^6^ Consequently, lower costs could be attributable to an earlier diagnosis and/or a milder degree of disability among PwMS without comorbidity, which is however not possible to observe with this study. Still, this assumption could be supported by our finding that PwMS without comorbidity had an average COI per patient lower than for any comorbidity group in the beginning of follow-up. On the other hand, GBTM revealed that a near-equal percentage of PwMS followed the trajectory with high healthcare costs and productivity losses in both the pooled population of PwMS with any comorbidity and the group of PwMS without comorbidity. This result indicates that other factors than comorbidity at the time of MS diagnosis may contribute to the heterogeneity in the development of COI of MS over time. Examples of such factors include: MS disease phenotype^28^ or the severity and activity grading of MS,^29^ which were not taken into account in this comparison. Further research is needed to assess the impact of comorbidities on the COI of MS including additional information such as MS subtype, severity, treatment protocol, and other parameters.

Secondary analysis of the trajectory model output revealed associations regarding the COI trajectories and sociodemographic characteristics with an emphasis on differences among those following any of the trajectories with high productivity losses. In general, PwMS who followed trajectories with high productivity losses were significantly older and the majority had at the most high-school level of education. This is in line with two previous studies which demonstrated that increased DP among older PwMS and that a lower educational level is associated with the absolute level and progression of work disability due to MS.^3,16^ We also found that significantly more women had trajectories with high productivity losses, which contradicts previous research. Despite MS being more prevalent in women,^1^ men in general have a higher COI of MS because of higher number of SA/DP days and a more rapid progression of MS.^30^ Due to the small sizes of the comorbidity groups, the results of the socio-demographic differences between the COI trajectories should be interpreted with caution.

### Study strengths and limitations

Several factors contributed to the strength of this first explorative study. The cohort design included the use of real-world longitudinal data linked at individual level from several population-based registers of high quality.^10,31^ This enabled the adoption of a societal perspective in estimating the COI of MS. Other strengths of the use of register data: not affected by self-reports; that all fulfilling the inclusion criteria, not a sample, could be included; that is, there were no drop-outs; that the large cohort allowed for sub-group analyses; that information on several covariates of importance for the outcomes could be included; the long follow-up time (7 years); and that all could be followed from diagnosis date, rather than by calendar year.

Lastly, the use of GBTM, which represents a novel approach in the examination of developmental trajectories of COI of MS enabled us to detect hidden developmental patterns of the outcome of interest that are not identified ex ante.^21^

The main limitation of this study lies in the fact that the comorbidity groups were defined at one point in time, in the period around the MS diagnosis date, in line with previous research regarding MS comorbidities.^32,33^ No follow-up regarding comorbidities was performed. Therefore, our study cannot depict the development of the impact comorbidities have on the COI of MS over time, since comorbidities at MS diagnosis may disappear over time, while new comorbidities can appear, changing the direction of the development of the COI of MS over time. Instead, we focused on the heterogeneity of the COI over time among people with MS categorized in different comorbidity groups.

Another limitation is the fact that comorbidity groupings, in some instances, were done due to prevalence but do not necessarily reflect similarities in the clinical manifestation of the diseases (e.g. grouping together cancer, cardiovascular disease, and genitourinary disease). Moreover, we did not study the differences between acute and chronic conditions. In addition, the study design did not allow to capture the interaction of individual comorbid conditions nor cumulative effect of multiple comorbidities on the COI trajectories.

Moreover, we did not account for the origin of comorbidities (whether diseases are related - due to - or unrelated to MS); the range of ICD-10 codes used for neurologic comorbidity encompasses multiple codes that may represent manifestations of MS which could lead to misclassification, and could explain the high prevalence of this comorbidity.

Due to data availability, information regarding primary healthcare was not available. This could underestimate the impact MS symptoms and comorbidities have on the COI in MS since some diagnoses related to MS symptoms or pharmacotherapy may mostly be treated there. In addition, we did not have information related to drugs administered within healthcare clinics. Moreover, costs of SA spells that were shorter (≤14 days), informal care, other non-healthcare costs, the costs of early mortality, and intangible costs were not included. Thus, the COI estimations may have been slightly underestimated. Furthermore, information on MS disability and its progression over time was not available in our data.

Lastly, our findings are generalisable to the healthcare and social insurance system as well as a working-age population of PwMS in Sweden. Thus, the generalisability may be limited to settings with differently organised systems and deviating employment frequencies.

## Conclusion

This register-based study showed that PwMS with comorbidities follow distinct COI trajectories over time. Heterogeneity in the COI, high healthcare costs and productivity losses, were observed over time, part of which could be explained by the presence of chronic comorbidities, such as mental and musculoskeletal disorders, at the time of MS diagnosis.

## Data statement

The data cannot be made publicly available due to privacy regulations. According to the General Data Protection Regulation, the Swedish Data Protection Act, the Swedish Ethical Review Act, and the Swedish Public Access to Information and Secrecy Act, data can only be made available for specific purposes, including research that meets the criteria for access to this type of sensitive and confidential data as determined by a legal review. Readers may contact Professor Kristina Alexanderson (kristina.alexanderson@ki.se) regarding the data.

## Conflict of Interests

CM and EF were partly funded by Biogen. EF has received an unrestricted research grant from Celgene. KA has received unrestricted researcher-initiated grants from Biogen. GB and AE conducted this work as part of their master’s thesis and have received funding by Biogen after the completion of their master thesis to draft this manuscript and present their results of their thesis. KK is only affiliated with Karolinska Institutet, not receiving financial compensation for her involvement in this study; she is working full time at Gilead Sciences AB. JH received honoraria for serving on advisory boards for Biogen and Novartis and speaker’s fees from Biogen, Merck-Serono, Bayer-Schering, Teva and Sanofi-Aventis. He has served as P.I. for projects sponsored by, or received unrestricted research support from, Biogen, Merck-Serono, TEVA, Novartis, and Bayer-Schering. His MS research is funded by the Swedish Research Council.

## Supplemental Material

sj-pdf-1-mso-10.1177_2055217320968597 - Supplemental material for Cost-of-illness trajectories among people with multiple sclerosis by comorbidity: A register-based prospective study in Sweden Click here for additional data file. Supplemental material, sj-pdf-1-mso-10.1177_2055217320968597 for Cost-of-illness trajectories among people with multiple sclerosis by comorbidity: A register-based prospective study in Sweden by Greta Bütepage, Ahmed Esawi, Kristina Alexanderson, Emilie Friberg, Chantelle Murley Jan Hillert Korinna Karampampa in Multiple Sclerosis Journal—Experimental, Translational and Clinical
