# Supplementary material for: Cost-of-illness trajectories among people with multiple sclerosis by comorbidity: A register-based prospective study in Sweden
Source: Mult Scler J Exp Transl Clin. 2020 Oct 23;6(4):2055217320968597. doi: 10.1177/2055217320968597 (PMC7585903; doi:10.1177/2055217320968597)
Supplement: sj-pdf-1-mso-10.1177_2055217320968597 - Supplemental material for Cost-of-illness trajectories among people with multiple sclerosis by comorbidity: A register-based prospective study in Sweden [file sj-pdf-1-mso-10.1177_2055217320968597.pdf]

Online supplementary material:

Table 1 (supplementary): Average healthcare costs and productivity losses, respectively, among people with MS , during all seven years of follow-up [95% confidence interval] in Euros 2018, by comorbidity and type of cost

	**Any comorbidity** N=499	**No comorbidity** N=140	**Ocular comorbidities (H00-H59)** N=169	**Cardiovascular, genitourinary or cancer disease (I00-I99, N00-N99, C00-D49)** N=125	**Musculoskeletal comorbidities (M00–M99)** N=74	**Mental comorbidities (F30–48, F50–59)** N=41	**Neurological comorbidities (G00–G99)** N=238	**Injuries (S00–T88)** N=52
**Healthcare costs**								
2006	8868 [8836-8911]	5526 [5498-5572]	9682 [9576-9747]	12,392 [12,264-12,510]	9285 [9144-9324]	10,604 [10,445-10,710]	9997 [9904-10,041]	11,231 [10,881-11,351]
2007	9420 [9411-9491]	7862 [7816-7908]	9890 [9843-9958]	12,397 [12,317-12,563]	9627 [9553-9713]	10,380 [10,273-10,492]	9332 [9266-9381]	8734 [8640-8806]
2008	8771 [8742-8811]	7509 [7461-7561]	8910 [8883-8980]	8929 [8873-8985]	11,355 [11,243-11,506]	11,393 [11,302-11,542]	8911 [8860-8963]	7844 [7737-7899]
2009	7436 [7406-7465]	7514 [7487-7579]	7362 [7330-7417]	8554 [8495-8613]	7221 [7150-7280]	8732 [8614-8822]	8450 [8388-8487]	7329 [7185-7366]
2010	7631 [7600-7663]	7901 [7862-7956]	8008 [7965-8066]	8377 [8317-8438]	8396 [8311-8484]	8853 [8726-8966]	8299 [8245-8352]	5776 [5720-5836]
2011	7618 [7580-7651]	7067 [7045-7139]	7743 [7683-7802]	9234 [9155-9302]	8488 [8407-8648]	8358 [8257-8495]	8179 [8132-8238]	5821 [5726-5872]
2012	7808 [7768-7844]	6757 [6735-6842]	8053 [7995-8107]	10,242 [10,148-10,356]	6645 [6558-6679]	8229 [8151-8331]	8188 [8137-8250]	6934 [6805-6990]
2013	7229 [7199-7264]	6992 [6962-7063]	8016 [7954-8062]	7223 [7151-7256]	5695 [5626-5737]	8147 [8049-8220]	8027 [7973-8086]	7268 [7136-7331]
**Productivity losses**								
2006	16,482 [16,426-16,547]	11,724 [11,646-11,873]	12,658 [12,598-12,773]	20,383 [20,276-20,525]	22,371 [22,215-22,540]	27,519 [27,258-27,685]	16,189 [16,088-16,266]	20,143 [19,939-20,308]
2007	18,342 [18,295-18,430]	13,477 [13,365-13,608]	15,268 [15,140-15,358]	21,401 [21,290-21,561]	22,130 [21,977-22,321]	28,389 [28,101-28,578]	17,960 [17,858-18,057]	21,503 [21,290-21,703]
2008	18,644 [18,594-18,732]	15,032 [14,902-15,159]	14,909 [14,771-14,989]	22,307 [22,181-22,468]	23,321 [23,117-23,469]	29,584 [29,292-29,777]	18,625 [18,525-18,728]	22,057 [21,866-22,274]
2009	18,791 [18,734-18,876]	16,070 [15,937-16,207]	14,904 [14,761-14,984]	21,763 [21,622-21,917]	22,712 [22,510-22,863]	27,741 [27,395-27,900]	19,036 [18,938-19,148]	22,652 [22,459-22,882]
2010	17,887 [17,832-17,972]	16,364 [16,216-16,481]	13,423 [13,288-13,503]	20,795 [20,656-20,951]	20,686 [20,509-20,846]	27,476 [27,110-27,630]	18,117 [18,020-18,224]	21,219 [21,031-21,462]
2011	17,185 [17,130-17,268]	16,510 [16,334-16,601]	13,786 [13,645-13,860]	20,048 [19,913-20,198]	17,876 [17,676-18,024]	25,404 [25,078-25,588]	17,814 [17,719-17,923]	21,361 [21,157-21,582]
2012	17,399 [17,322-17,463]	16,545 [16,371-16,643]	14,717 [14,583-14,807]	20,109 [19,997-20,292]	16,734 [16,542-16,899]	25,927 [25,612-26,150]	17,811 [17,719-17,927]	21,944 [21,747-22,187]
2013	17,882 [17,807-17,951]	16,794 [16,624-16,906]	14,170 [14,036-14,261]	20,566 [20,446-20,752]	17,817 [17,648-18,011]	25,107 [24,758-25,324]	18,680 [18,602-18,814]	21,699 [21,504-21,942]
**Total costs**								
2006	25,351 [25,287-25,434]	17,251 [14,119-20,382]	22,341 [19,079-25,603]	32,776 [28,230-37,322]	31,656 [26,728-36,584]	38,124 [30,705-45,542]	26,187 [23,292-29,081]	31,375 [23,975-38,776]

2007	27,762 [27,732-27,896]	21,339 [18,125-24,552]	25,158 [21,925-28,391]	33,798 [28,850-38,746]	31,757 [26,880-36,635]	38,770 [31,344-46,195]	27,293 [24,211-30,375]	30,237 [24,509-35,966]
2008	27,416 [27,360-27,520]	22,542 [19,098-25,986]	23,820 [20,740-26,900]	31,237 [27,375-35,099]	34,677 [28,618-40,736]	40,977 [33,672-48,282]	27,536 [24,492-30,581]	29,902 [24,095-35,710]
2009	26,228 [26,162-26,321]	23,584 [19,958-27,211]	22,266 [19,192-25,341]	30,317 [26,193-34,442]	29,933 [25,184-34,683]	36,474 [28,987-43,961]	27,486 [24,377-30,596]	29,981 [23,698-36,264]
2010	25,519 [25,454-25,613]	24,266 [20,694-27,837]	21,432 [18,316-24,549]	29,173 [25,025-33,321]	29,083 [24,005-34,161]	36,330 [28,638-44,023]	26,417 [23,319-29,515]	26,996 [21,114-32,877]
2011	24,818 [24,746-24,912]	23,578 [19,916-27,239]	21,529 [18,106-24,952]	29,283 [24,903-33,663]	26,476 [20,457-32,495]	33,763 [25,914-41,611]	25,994 [22,883-29,105]	27,286 [21,418-33,154]
2012	25,220 [25,124-25,298]	23,302 [19,570-27,034]	22,770 [19,347-26,193]	30,352 [25,290-35,414]	23,448 [18,290-28,606]	34,156 [26,180-42,133]	26,000 [22,814-29,187]	28,980 [22,727-35,233]
2013	25,119 [25,039-25,200]	23,786 [19,941-27,631]	22,186 [18,974-25,399]	27,790 [23,703-31,876]	23,551 [18,597-28,505]	33,255 [25,239-41,272]	26,708 [23,559-29,856]	29,055 [23,045-35,065]

[1]From people with MS that met the inclusion criteria for our study, only 499 of those had at least one relevant comorbidity at the time of MS diagnosis, i.e. they were belonging in at least one of the defined six comorbidity groups, or had no comorbidity (n=140). Therefore, results in this table are presented for 639 people with MS in total. Since PwMS can present with multiple comorbidities, they can be included in more than one comorbidity group (out of these six comorbidity groups).

Table 2 (supplementary): Average costs of cost components among 639 people with MS, during all seven years of follow-up [95% confidence interval] in Euros 2018, by comorbidity, and type of cost (a-b)

a) Healthcare costs

	Any comorbidity N=499	No comorbidity N=140	Ocular comorbidities (H00-H59) N=169	Cardiovascular, genitourinary or cancer disease (I00–I99, N00–N99, C00–D49) N=125	Musculoskeletal comorbidities (M00–M99) N=74	Mental comorbidities (F30–48, F50–59) N=41	Neurological comorbidities (G00–G99) N=238	Injuries (S00–T88) N=52
**Inpatient healthcare costs**								
2006	3324 [3290-3350]	756 [746-770]	3343 [3261-3406]	5615 [5494-5701]	4391 [4283-4436]	4787 [4625-4858]	4275 [4202-4318]	6229 [5913-6339]
2007	2189 [2179-2243]	477 [459-485]	2115 [2086-2155]	4058 [3990-4223]	3182 [3123-3253]	2420 [2339-2468]	1794 [1742-1833]	1465 [1432-1516]
2008	2082 [2057-2101]	915 [877-936]	1730 [1712-1771]	1780 [1758-1815]	5321 [5226-5472]	3398 [3312-3479]	1893 [1874-1923]	2390 [2330-2422]
2009	1314 [1301-1331]	933 [911-957]	893 [878-915]	1849 [1817-1902]	1406 [1365-1447]	1678 [1571-1707]	1681 [1651-1703]	1897 [1811-1915]
2010	1836 [1814-1852]	1513 [1471-1531]	1888 [1853-1920]	1888 [1868-1941]	2831 [2777-2916]	1872 [1763-1950]	2062 [2038-2103]	1155 [1122-1186]
2011	1718 [1692-1739]	1100 [1070-1123]	1609 [1567-1642]	2081 [2052-2132]	2566 [2512-2734]	1915 [1813-2014]	1914 [1887-1954]	1580 [1507-1600]
2012	2145 [2112-2168]	1022 [997-1063]	1945 [1906-1983]	3814 [3744-3935]	1173 [1144-1205]	1896 [1825-1943]	2208 [2178-2249]	3032 [2905-3062]
2013	1469 [1458-1488]	912 [878-928]	1329 [1303-1357]	1313 [1283-1334]	501 [479-514]	1767 [1693-1777]	1986 [1966-2019]	2861 [2739-2891]
**Copayments - Inpatient healthcare**								
2006	32 [32-33]	11 [11-11]	29 [29-30]	45 [45-46]	43 [42-43]	49 [48-49]	35 [35-36]	44 [44-45]
2007	18 [18-18]	5 [5-5]	19 [19-20]	25 [25-26]	27 [26-27]	27 [26-27]	16 [16-16]	13 [13-14]
2008	14 [14-14]	5 [5-5]	12 [12-13]	13 [13-13]	27 [26-27]	32 [31-33]	14 [14-14]	19 [18-19]
2009	10 [10-11]	5 [5-5]	8 [8-8]	11 [11-11]	10 [10-10]	27 [26-28]	12 [11-12]	21 [20-21]
2010	12 [12-12]	13 [12-13]	13 [13-13]	13 [13-14]	16 [16-17]	11 [10-11]	12 [12-12]	12 [11-12]
2011	11 [11-12]	7 [7-7]	11 [10-11]	16 [16-17]	9 [8-9]	10 [10-10]	12 [12-13]	16 [15-16]
2012	13 [12-13]	6 [6-6]	13 [13-13]	18 [18-19]	6 [6-6]	20 [19-20]	13 [13-13]	18 [17-18]
2013	11 [11-11]	6 [6-6]	8 [8-8]	11 [11-11]	2 [2-2]	21 [20-21]	14 [14-14]	17 [16-17]
**Outpatient healthcare costs**								
2006	1995 [1988-2004]	808 [803-815]	2181 [2162-2187]	2914 [2897-2951]	2252 [2221-2259]	2396 [2359-2412]	2122 [2109-2132]	2304 [2269-2326]

2007	1165 [1158-1172]	538 [533-541]	1379 [1364-1398]	1591 [1574-1607]	1213 [1203-1223]	1569 [1546-1584]	1091 [1085-1098]	1647 [1607-1661]
2008	1120 [1116-1126]	541 [536-545]	1161 [1151-1170]	1310 [1300-1324]	1059 [1048-1067]	1414 [1400-1427]	1082 [1077-1087]	1339 [1317-1353]
2009	1173 [1168-1180]	831 [824-841]	1122 [1113-1130]	1346 [1336-1363]	1308 [1290-1322]	1398 [1376-1413]	1329 [1319-1337]	1566 [1530-1586]
2010	1120 [1116-1129]	702 [693-706]	1150 [1141-1163]	1469 [1456-1485]	1125 [1106-1135]	1440 [1408-1446]	1209 [1202-1220]	933 [922-943]
2011	1261 [1253-1265]	763 [756-767]	1188 [1177-1194]	1642 [1628-1656]	1237 [1217-1244]	1198 [1169-1207]	1401 [1392-1411]	1104 [1091-1114]
2012	1341 [1337-1347]	931 [923-939]	1408 [1398-1417]	1567 [1554-1578]	1349 [1322-1349]	1543 [1524-1560]	1370 [1363-1379]	1333 [1320-1349]
2013	1396 [1389-1401]	1015 [1008-1023]	1420 [1407-1428]	1556 [1540-1569]	1551 [1537-1569]	1734 [1701-1750]	1404 [1390-1407]	1520 [1502-1536]
**Copayments - Outpatient healthcare**								
2006	108 [108-109]	64 [63-64]	115 [114-115]	110 [110-111]	112 [111-112]	113 [113-113]	110 [110-110]	104 [104-105]
2007	78 [78-78]	50 [49-50]	77 [77-77]	84 [84-85]	85 [85-85]	91 [91-92]	81 [80-81]	87 [86-87]
2008	71 [71-71]	41 [41-42]	71 [71-72]	76 [76-76]	69 [69-70]	87 [86-87]	73 [73-73]	78 [77-78]
2009	61 [61-61]	44 [43-44]	61 [61-61]	67 [67-68]	65 [64-65]	75 [75-76]	64 [64-65]	69 [69-70]
2010	58 [58-58]	43 [43-43]	61 [61-62]	64 [64-64]	61 [61-62]	69 [68-69]	57 [57-58]	63 [63-63]
2011	59 [58-59]	46 [46-46]	59 [59-60]	68 [68-69]	60 [60-60]	57 [56-57]	61 [61-61]	62 [61-62]
2012	62 [61-62]	48 [48-48]	64 [63-64]	68 [67-68]	63 [62-63]	74 [73-74]	61 [61-61]	63 [62-63]
2013	61 [61-61]	51 [50-51]	63 [62-63]	62 [62-63]	66 [66-66]	70 [69-70]	61 [61-61]	66 [66-67]
**Drug costs**								
2006	3406 [3398-3431]	3886 [3858-3927]	4013 [3979-4037]	3705 [3673-3742]	2486 [2445-2511]	3258 [3232-3341]	3453 [3421-3468]	2548 [2498-2586]
2007	5968 [5954-6000]	6791 [6752-6841]	6298 [6262-6341]	6636 [6582-6681]	5118 [5061-5176]	6271 [6210-6380]	6348 [6311-6380]	5520 [5442-5583]
2008	5482 [5464-5515]	6004 [5973-6057]	5934 [5905-5983]	5748 [5693-5785]	4877 [4813-4927]	6460 [6407-6579]	5847 [5801-5884]	4017 [3945-4071]
2009	4876 [4848-4896]	5699 [5676-5756]	5276 [5247-5321]	5278 [5221-5308]	4430 [4372-4480]	5552 [5500-5661]	5362 [5315-5394]	3774 [3705-3820]
2010	4603 [4580-4628]	5629 [5611-5691]	4893 [4862-4937]	4942 [4881-4964]	4361 [4297-4403]	5460 [5404-5560]	4957 [4905-4985]	3612 [3562-3668]
2011	4581 [4558-4607]	5149 [5140-5218]	4874 [4841-4920]	5423 [5359-5455]	4718 [4655-4765]	5176 [5129-5284]	4789 [4747-4828]	3152 [3107-3208]
2012	4259 [4237-4285]	4748 [4732-4810]	4622 [4582-4659]	4773 [4718-4799]	4128 [4062-4167]	4694 [4650-4790]	4535 [4493-4573]	2598 [2558-2656]
2013	4301 [4276-4328]	5006 [4992-5078]	5194 [5145-5231]	4279 [4225-4304]	3632 [3576-3668]	4553 [4509-4655]	4560 [4517-4606]	2920 [2874-2986]

b) Productivity losses

	Any comorbidity N=499	No comorbidity N=140	Ocular comorbidities (H00-H59) N=169	Cardiovascular, genitourinary or cancer disease (I00–I99, N00–N99, C00–D49) N=125	Musculoskeletal comorbidities (M00–M99) N=74	Mental comorbidities (F30–48, F50–59) N=41	Neurological comorbidities (G00–G99) N=238	Injuries (S00–T88) N=52
**Disability pension costs**								
2006	7411 [7352-7459]	5231 [5173-5353]	3649 [3595-3720]	10,206 [10,094-10,342]	9790 [9696-10,011]	17,920 [17,585-18087]	6277 [6187-6333]	10,051 [9804-10,164]
2007	9072 [9010-9126]	7679 [7577-7791]	5221 [5156-5311]	12,167 [12,057-12,320]	11,962 [11,857-12,191]	17,712 [17,391-17,907]	8367 [8275-8440]	12,884 [12,632-13,006]
2008	11,451 [11,394-11,522]	10,428 [10,292-10,529]	7303 [7225-7405]	14,866 [14,746-15,023]	14,929 [14,793-15,148]	21,000 [20,705-21,235]	11,123 [11,033-11,219]	14,980 [14,725-15,138]
2009	13,510 [13,448-13,583]	13,107 [12,967-13,224]	9200 [9109-9306]	17,533 [17,378-17,670]	16,360 [16,185-16,543]	24,001 [23,665-24,192]	13,574 [13,476-13,671]	15852 [15,592-16,018]
2010	15,184 [15,124-15,263]	14,676 [14,526-14,792]	10,692 [10,577-10,785]	18,905 [18,762-19,064]	16,519 [16,350-16,694]	25,237 [24,853-25,392]	15,272 [15,162-15,366]	18,271 [18,068-18,509]
2011	14,682 [14,621-14,757]	14,574 [14,405-14,669]	10,591 [10,472-10,677]	17,767 [17,636-17,924]	15,251 [15,060-15,403]	23,894 [23,561-24,091]	15,101 [14,997-15,197]	18,607 [18,389-18,818]
2012	14,736 [14,670-14,808]	14,761 [14,600-14,868]	10,317 [10,196-10,401]	17,668 [17,550-17,844]	14,833 [14,659-15,006]	24,571 [24,241-24,793]	15,452 [15,353-15,557]	18,968 [18,738-19,177]
2013	15,185 [15,112-15,254]	14,858 [14,697-14,975]	10,600 [10,480-10,688]	18,471 [18,346-18,650]	15,504 [15,347-15,704]	24,635 [24,280-24,834]	15,993 [15,918-16,128]	18,908 [18,685-19,127]
**Sickness absence costs**								
2006	9071 [9035-9126]	6492 [6413-6579]	9008 [8954-9101]	10,177 [10,087-10,276]	12,580 [12,385-12,662]	9598 [9465-9806]	9912 [9847-9987]	10,092 [9993-10,285]
2007	9270 [9242-9346]	5797 [5724-5879]	10,046 [9923-10,106]	9233 [9138-9336]	10,168 [9989-10,259]	10,676 [10,481-10,899]	9593 [9523-9677]	8619 [8528-8826]
2008	7192 [7158-7252]	4604 [4547-4691]	7605 [7483-7646]	7441 [7344-7536]	8391 [8201-8443]	8583 [8378-8750]	7502 [7428-7572]	7077 [7007-7269]
2009	5280 [5249-5331]	2962 [2920-3031]	5703 [5591-5736]	4229 [4174-4316]	6352 [6211-6433]	3740 [3605-3832]	5461 [5406-5531]	6800 [6730-7001]
2010	2702 [2681-2735]	1688 [1651-1726]	2731 [2670-2757]	1889 [1848-1932]	4166 [4068-4241]	2239 [2159-2335]	2844 [2815-2899]	2947 [2861-3053]
2011	2503 [2483-2536]	1936 [1888-1973]	3194 [3126-3229]	2281 [2224-2326]	2624 [2541-2695]	1510 [1428-1585]	2713 [2684-2764]	2754 [2689-2842]
2012	2663 [2625-2681]	1783 [1729-1817]	4400 [4330-4462]	2441 [2396-2499]	1901 [1829-1946]	1355 [1289-1438]	2359 [2331-2404]	2976 [2923-3095]
2013	2696 [2668-2723]	1935 [1886-1972]	3570 [3507-3621]	2094 [2054-2146]	2313 [2237-2371]	472 [455-512]	2687 [2643-2726]	2790 [2732-2901]

Figure 1 (supplementary): Trend of average annual cost of illness (COI) per patient by comorbidity during the seven-year follow-up period

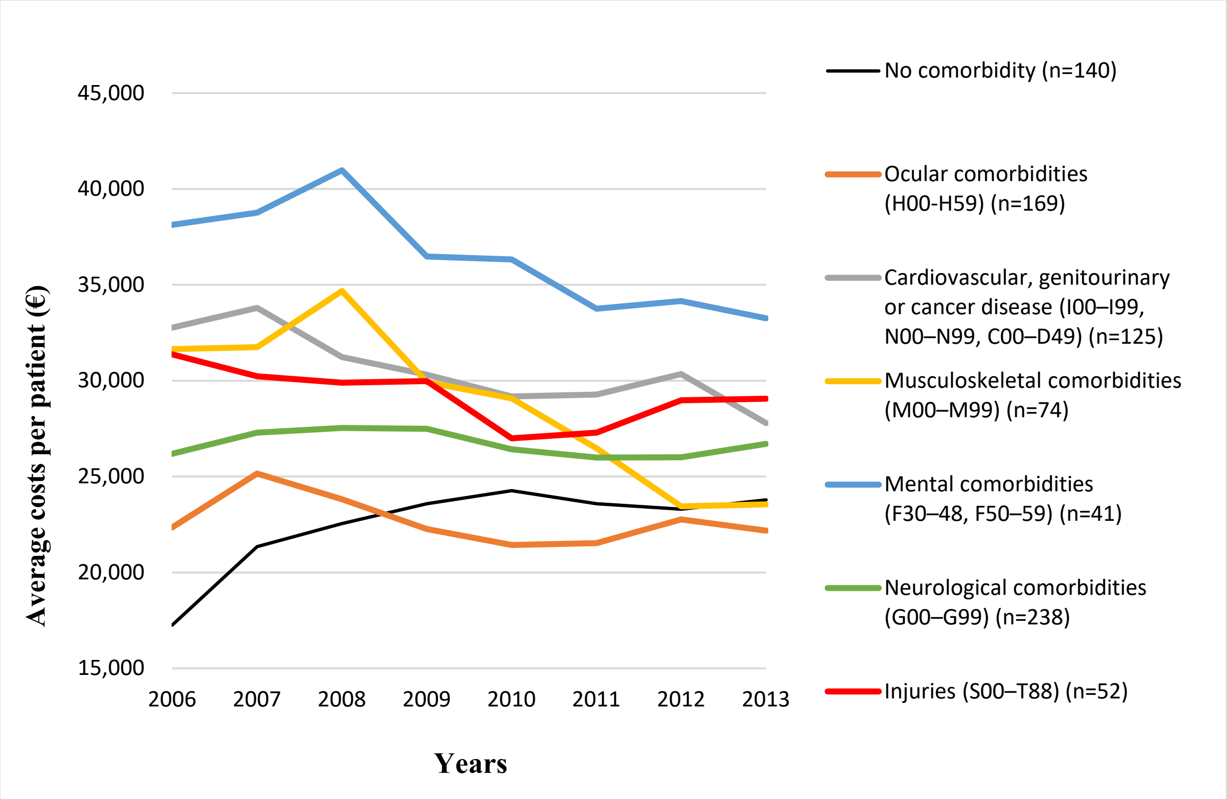

Figure 2 a–g (supplementary): Trajectories of healthcare cost (HC) and productivity losses (PL), respectively, among people with MS by comorbidity (the dotted lines represent 95% confidence intervals) over the future seven years from date when diagnosed with MS in 2006

a) Healthcare costs and productivity loss trajectories of PwMS with ocular comorbidities (n=169)

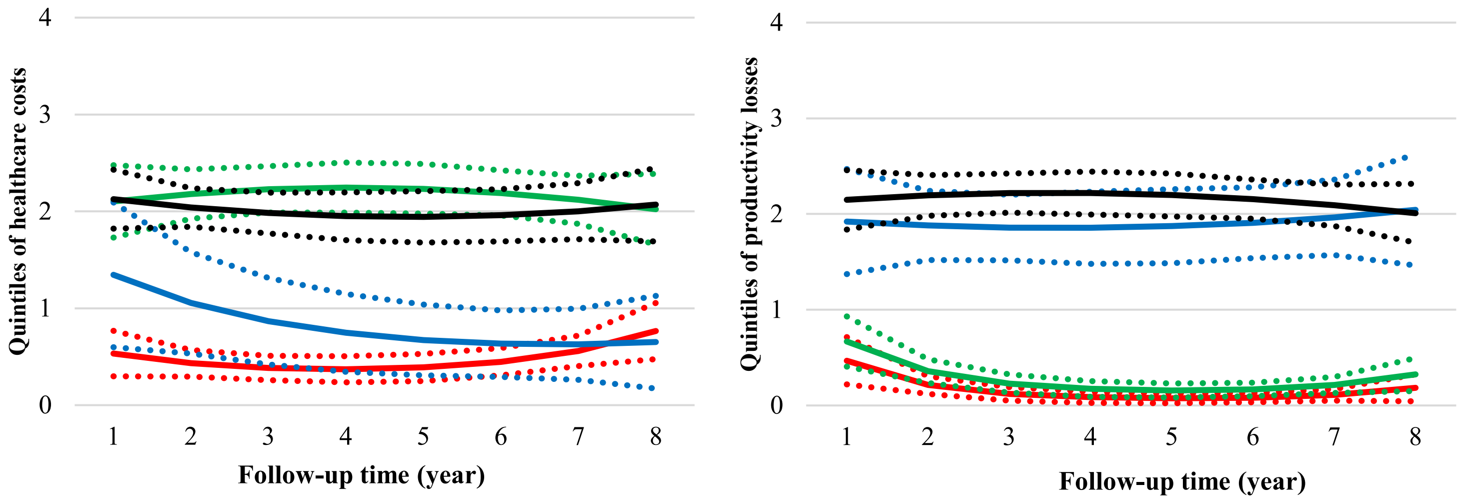

Trajectory 1; low healthcare costs and productivity losses (19.1% of all PwMS with ocular comorbidities)

Trajectory 2; high healthcare costs but low productivity losses (27.2%)

Trajectory 3; low healthcare costs but high productivity losses (13.1%)

Trajectory 4; high healthcare costs and productivity losses (40.6%)

b) Healthcare costs and productivity loss trajectories of PwMS with neurological comorbidities (n=238)

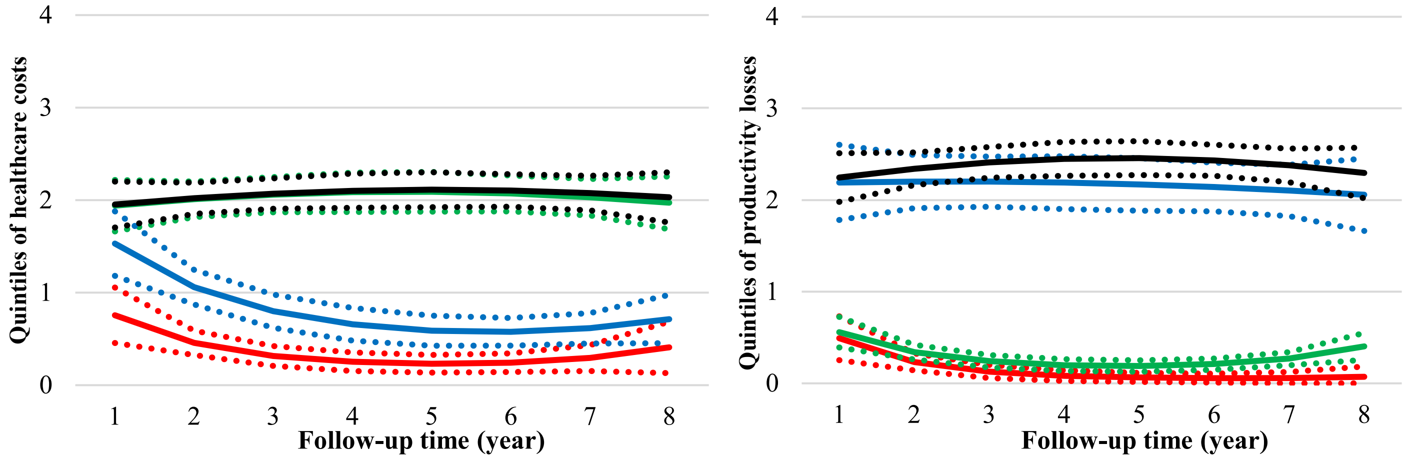

Trajectory 1; low healthcare costs and productivity losses (13.2% of all PwMS with neurological comorbidities)

Trajectory 2; high healthcare costs but low productivity losses (29.5%)

Trajectory 3; low healthcare costs but high productivity losses (19.1%)

Trajectory 4; high healthcare costs and productivity losses (38.2%)

c) Healthcare costs and productivity loss trajectories for PwMS with cardiovascular, genitourinary, or cancer disease (n=125)

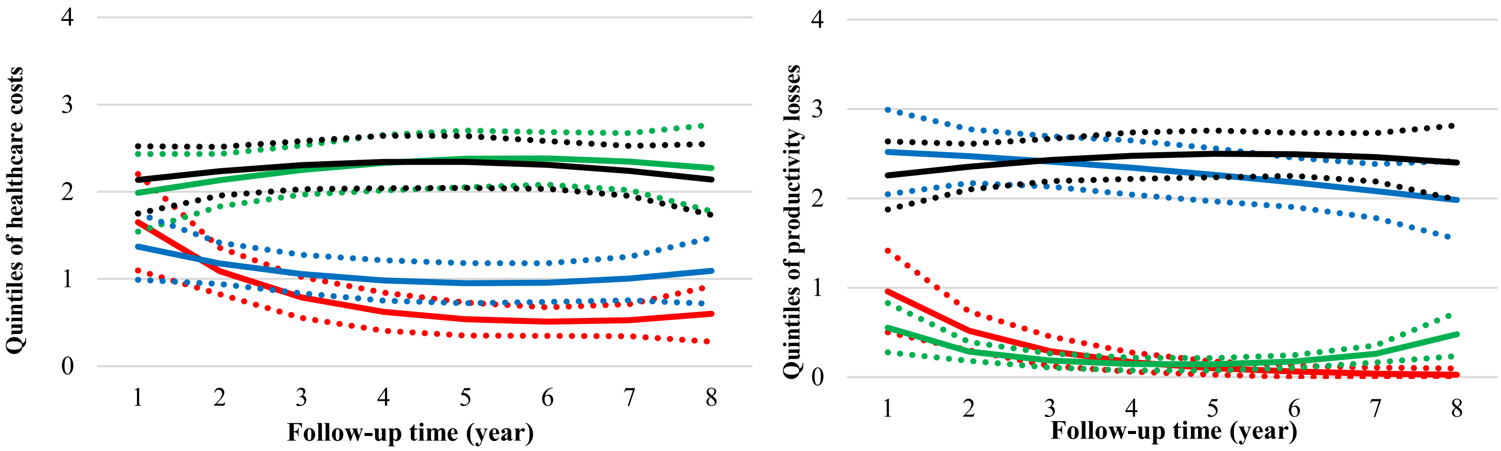

Trajectory 1; low healthcare costs and productivity losses (14.5% of all PwMS with cardiovascular, genitourinary, or cancer disease)

Trajectory 2; high healthcare costs but low productivity losses (22.1%)

Trajectory 3; low healthcare costs but high productivity losses (27.1%)

Trajectory 4; high healthcare costs and productivity losses (36.3%)

d) Healthcare costs and productivity loss trajectories of PwMS with injuries (n=52)

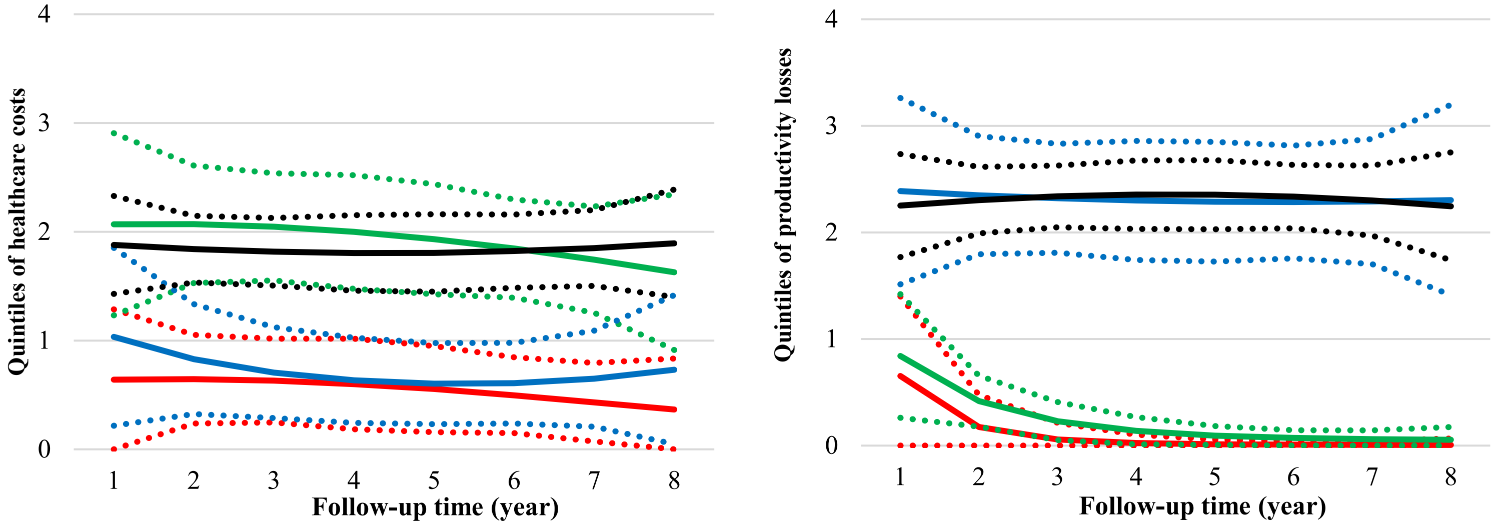

Trajectory 1; low healthcare costs and productivity losses (9.2% of all PwMS with injuries)

Trajectory 2; high healthcare costs but low productivity losses (17.7%)

Trajectory 3; low healthcare costs but high productivity losses (18.5%)

Trajectory 4; high healthcare costs and productivity losses (54.6%)

e) Healthcare costs and productivity loss trajectories for PwMS with mental comorbidities (n=41)

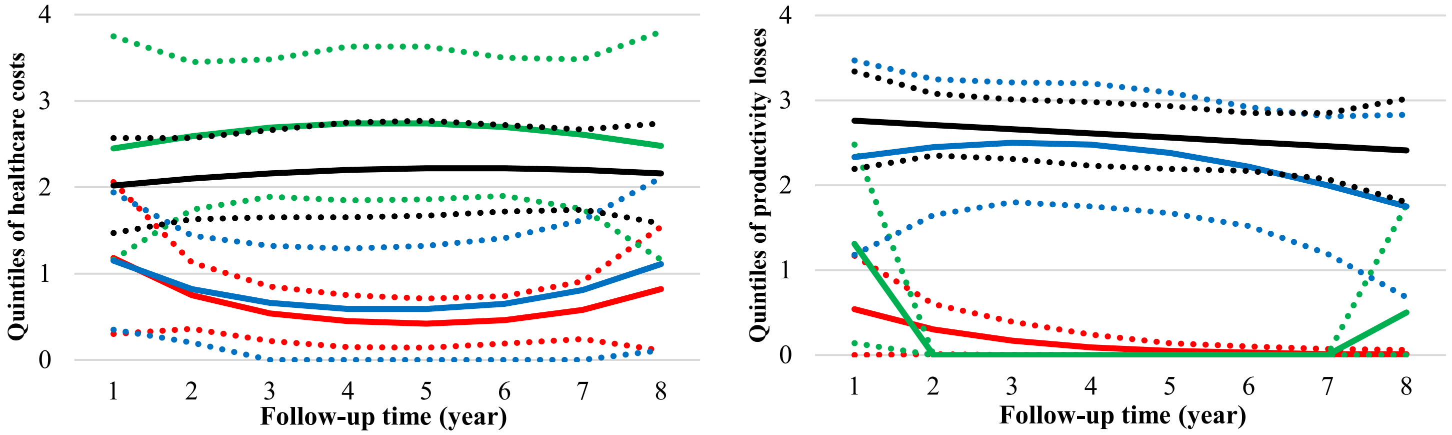

Trajectory 1; low healthcare costs and productivity losses (12.2% of all PwMS with mental comorbidities)

Trajectory 2; high healthcare costs but low productivity losses (10.1%)

Trajectory 3; low healthcare costs but high productivity losses (18%)

Trajectory 4; high healthcare costs and productivity losses (59.6%)

f) Healthcare costs and productivity loss trajectories of PwMS with musculoskeletal comorbidities (n=74)

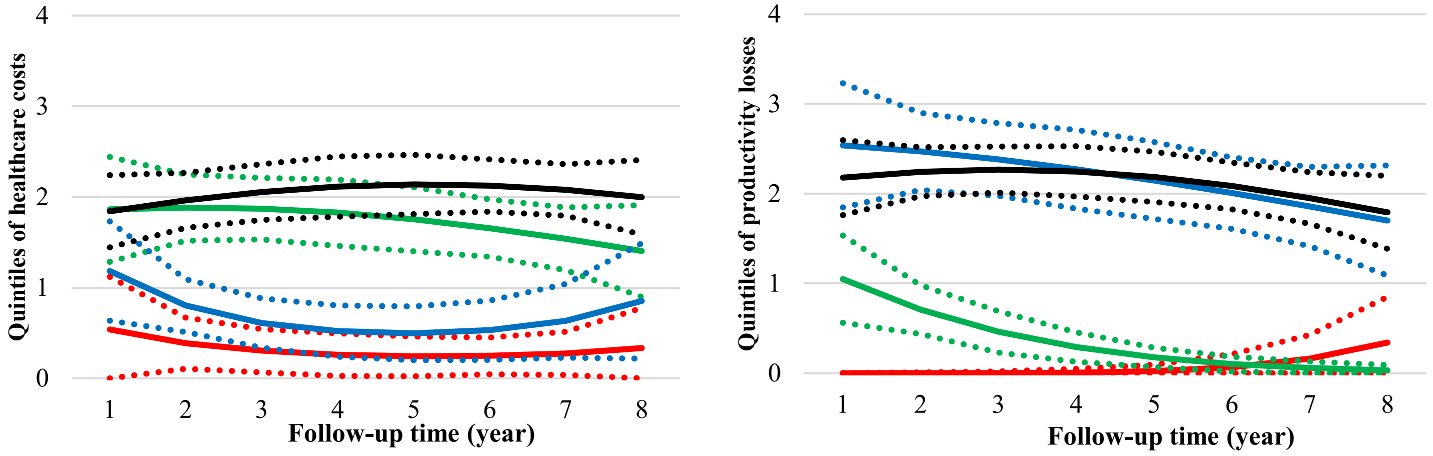

Trajectory 1; low healthcare costs and productivity losses (6.7% of all PwMS with musculoskeletal comorbidities)

Trajectory 2; high healthcare costs but low productivity losses (21.6%)

Trajectory 3; low healthcare costs but high productivity losses (21.5%)

Trajectory 4; high healthcare costs and productivity losses (50.2%)
